# Evaluation of C-reactive protein, Haptoglobin and cardiac troponin 1 levels in brachycephalic dogs with upper airway obstructive syndrome

**DOI:** 10.1186/1746-6148-8-152

**Published:** 2012-08-31

**Authors:** Marta Planellas, Rafaela Cuenca, Maria-Dolores Tabar, Coralie Bertolani, Cyrill Poncet, Josep M Closa, Juan Lorente, Jose J Cerón, Josep Pastor

**Affiliations:** 1 Animal Medicine and Surgery Department. Faculty of Veterinary Medicine, Universitat Autònoma de Barcelona (UAB), Cerdanyola del Vallès, Barcelona, 08193, Spain; 2 Centro Policlínico Veterinario Raspeig, Camino Rodalet nº 17, Sant Vicente del Raspeig, Alicante, 03690, Spain; 3 Centre Hospitalier Vétérinaire Frégis, Av. Aristide Briand nº 43, Arcueil, 94110, France; 4 Hospital ARS Veterinària, Cardedeu str. nº 3, Barcelona, 08023, Spain; 5 Otorrinolaringology service in the Hospital General Universitari de la Vall d’Hebron, Passeig de la Vall d’Hebron nº 119-129, Barcelona, 08035, Spain; 6 Department of Animal Medicine and Surgery, Faculty of Veterinary Medicine, Regional Campus of International Excellence "Campus Mare Nostrum" Murcia University, Espinardo, Murcia, 30100, Spain

**Keywords:** Acute phase proteins, Apnea, Brachycephalic, Hypoxia, Myocardial damage, Upper airway

## Abstract

**Background:**

Brachycephalic dogs have unique upper respiratory anatomy with abnormal breathing patterns similar to those in humans with obstructive sleep apnea syndrome (OSAS). The objective of this study was to evaluate the correlation between anatomical components, clinical signs and several biomarkers, used to determine systemic inflammation and myocardial damage (C-reactive protein, CRP; Haptoglobin, Hp; cardiac troponin I, cTnI), in dogs with brachycephalic upper airway obstructive syndrome (BAOS).

**Results:**

Fifty brachycephalic dogs were included in the study and the following information was studied: signalment, clinical signs, thoracic radiographs, blood work, ECG, components of BAOS, and CRP, Hp and cTnI levels. A high proportion of dogs with BAOS (88%) had gastrointestinal signs. The prevalence of anatomic components of BAOS was: elongated soft palate (100%), stenotic nares (96%), everted laryngeal saccules (32%) and tracheal hypoplasia (29.1%). Increased serum levels of biomarkers were found in a variable proportion of dogs: 14% (7/50) had values of CRP > 20 mg/L, 22.9% (11/48) had values of Hp > 3 g/L and 47.8% (22/46) had levels of cTnI > 0.05 ng/dl. Dogs with everted laryngeal saccules had more severe respiratory signs (p<0.02) and higher values of CRP (p<0.044). No other statistical association between biomarkers levels and severity of clinical signs was found.

**Conclusions:**

According to the low percentage of patients with elevated levels of CRP and Hp, BAOS does not seem to cause an evident systemic inflammatory status. Some degree of myocardial damage may occur in dogs with BAOS that can be detected by cTnI concentration.

## Background

Brachycephalic upper airway obstructive syndrome (BAOS) is a combination of nasal and oropharyngeal anatomic abnormalities resulting from selective breeding to reduce the length of the maxilla without concurrent reduction in the soft tissue of the nose, palate, and pharynx [1,2]. The resulting excess of soft tissue causes airway obstruction in affected animals, with clinical signs that may include inspiratory stertor and stridor, exercise and heat intolerance, cyanosis, respiratory distress, regurgitation, and vomiting. The primary abnormalities of BAOS are stenotic nares and an overlong soft palate. Secondary changes that occur as a result of chronic upper airway obstruction include eversion of laryngeal saccules, pharyngeal edema and laryngeal collapse. Brachycephalic dogs can present other respiratory tract abnormalities such as tracheal hypoplasia, bronchial collapse, macroglossia, reduced buccal opening, nasopharyngeal collapse and nasopharyngeal turbinates [1-7]. Such dogs have anatomic abnormalities that include small pharyngeal airways and abnormal breathing patterns similar to those in humans with obstructive sleep apnea syndrome (OSAS) [8,9]. On the other hand, it have been reported that the English bulldog has snoring, fragmented sleep with mild hypoxemia and apneas in the same manner as human patients with OSAS. Those anatomic and clinical traits, made the English bulldog a suitable natural animal model for human OSAS [8,9].

Inflammation is the characteristic trait of the pathophysiology of OSAS and represents a pathway linking human OSAS patients with increased cardiovascular morbidity [10,11]. C-reactive protein (CRP) levels have been shown to be high in the presence of hypoxia, for example at high altitude [12]. Furthermore, CRP may also increase with sleep deprivation [13]. Both hypoxemia and sleep deprivation are frequently present in human patients with OSAS, and therefore may participate in the genesis of an inflammatory response [13]. Hypoxia also may increase fatigability of upper airway dilator muscle and soft tissue, in a manner that has the potential to worsen disease. Inflammation, as well as oxidative stress, resulted from the repeated hypoxia and re-oxygenations could be implicated in the injury to these tissues [14,15].

Some epidemiological studies have linked high haptoglobin (Hp) levels with a greater incidence of myocardial infarct [16]. Kim and others [16] have reported that human patients with OSAS have higher levels of Hp, which fell after medical treatment with a continuous positive airway pressure device.

Cardiac troponin I (cTnI) and T (cTnT) are definitive biomarkers for detection of myocardial injury in human patients [17,18], and more recently in dogs [19,20]. In dogs with acute myocardial injuries or infarction, the concentration of cTnI has been shown to be positively correlated with the size of the infarct [21]. Moreover, chronic increases in cTnI are associated with chronic or ongoing pathologic myocardial process [21].

Even though human OSAS is not equal to canine BAOS, similarities exist. On the other hand, dogs with BAOS present clinical abnormalities (chronic hypoxia, gastrointestinal signs and subclinical heatstroke) that make it worthy to rule out the presence of systemic inflammation and myocardial damages.

The objective of this study was to evaluate the correlation between anatomical components, clinical signs and several biomarkers, used to determine systemic inflammation and myocardial damage (CRP, Hp, cTnI), in dogs with BAOS.

## Results

### Epidemiological data

Male dogs were over-represented in the study since 30 males (two neutered) and 20 females (four neutered) were evaluated. The median age was 2.5 years old (range 1–10 years old). Thirty-eight dogs (76%) were less than three years old. All were purebred: French bulldogs were the commonest breed studied and accounted for 29 of the 50 cases (58%), while other breeds evaluated included English bulldogs (n = 8, 16%), Pug (n = 12, 24%) and Shih-tzu (n = 1, 2%).

### Clinical signs

All dogs had some kind of respiratory signs (Table 1): only one dog had a mild grade of respiratory signs (grade 1), 13 (26%) suffered from a moderate grade (grade 2) and 36 (72%) from a severe grade (grade 3). Although a large number of animals with respiratory signs also had digestive signs (mild or moderate), no correlation was found between both types of signs (*p* = 0.71).

**Table 1:** Gradation of respiratory and digestive signs in 50 brachycephalic dogs suffering from BAOS

**Grade of clinical signs**	**Respiratory signs**		**Grade of clinical signs**	**Digestive signs**	
	**(stertors, snoring, inspiratory efforts, stress, exercise intolerance, syncope)**			**(regurgitation, vomiting)**	
	**n**	**%**		**n**	**%**
**Grade 0- Absent**	0	0%	**Grade 0- Absent**	6	12%
**Grade 1-Mild Stertors during exercise** (<once week)	1	2%	**Grade 1-Mild** (< once week)	23	46%
**Grade 2-Moderate Continuous stertor** (< once daily)	13	26%	**Grade 2-Moderate** (< once daily)	14	28%
**Grade 3-Severe Exercice intolerance, cianosis** (>once daily)	36	72%	**Grade 3-Severe** (> once daily)	7	14%

Electrocardiograms (ECG) were recorded previously to upper airway (UA) inspection and the presence of arrhythmia and heart rate was recorded. Three out of fifty presented sinusal arrhythmia indicating parasympathetic dominance. Heart rate values ranged from 70 to 180 beats per minute. No other abnormalities have been observed in ECG reports.

The inspection of the UA respiratory tracts revealed abnormalities in all 50 patients: abnormally long and hyperplasic soft palate (50/50 dogs; 100%), stenotic nares (48/50 dogs; 96%) and everted laryngeal saccules (16/50 dogs; 32%). Thoracic radiography was performed in 48 cases and tracheal hypoplasia was diagnosed in 14 animals 29.1% dogs; pug (n = 4), English bulldog (n = 2) and French bulldog (n = 8)] with a DTI/DT (thoracic inlet to tracheal diameter) ratio that ranged from 0.08 to 0.3. Thoracic radiography also allowed us to record vertebral heart score (VHS) of 35 out of 50 patients, obtaining values from 8.5 to 12.1 [22]. The elevated incidence of hemivertebra in brachycephalic dogs made the VHS measurement impossible in 15 dogs.

### Laboratory data

Table 2 shows the relationship between CRP, Hp and cTnI levels in the different breeds. The median CRP concentration in brachycephalic dogs with BAOS was 7.8 mg/L (3.6-235 mg/L). Seven of the 50 dogs studied had high concentrations of CRP. Hp levels were measured in 48 dogs, with a median Hp concentration of 1.6 g/L (range 0.09-13.9 g/L). From those, 11 (22.9%) had increased Hp levels. The median cTnI value was 0.05 ng/ml (<0.05–0.29), while 47.8% (22/46) of dogs with BAOS had high values of cTnI (>0.05).

**Table 2:** CRP, Hp and cTnI levels in the different breeds of the 50 brachycephalic dogs studied

**Breed**	**CRP > 20 mg/dl**		**Hp > 3 g/L**		**cTnI > 0.05 ng/dl**	
	**n**	**%**	**n**	**%**	**n**	**%**
**English B**	3/8	37.5	0/8	0	8/8	100
**French B**	3/29	10.3	5/27	18.5	14/26	53.8
**Pug**	1/12	8.3	6/12	50	0/11	0
**Shitzu**	0/1	0	0/1	0	0/1	0
**Total**	7/50	14	11/48	22.9	22/46	47.8

Table 3 shows that there was a significant correlation between the severity of the respiratory signs and male patients (*p* = 0.026).

**Table 3:** Statistical analysis showing the association of different variables with respiratory and digestive signs

**Variable**	**Respiratory signs**		**Digestive signs**	
	**∂**	***p *value**	**∂**	***p *value**
**Age**	0.154	0.255	0.052	0.645
**Sex**	- 0.317*	0.026*	- 0.162	0.258
**Breed**	0.197	0.17	- 0.254	0.067
**HTC**	- 0.003	0.990	- 0.131	0.99
**WBC**	- 0.099	0.583	- 0.016	0.916
**Platelets**	0.008	0.97	- 0.039	0.8
**Total Protein**	0.347	0.066	0.206	0.28
**Ratio DT/DTI**	- 0.187	0.195	- 0.127	0.38
**CRP**	0.076	0.581	- 0.063	0.66
**Hp**	- 0.025	0.87	- 0.143	0.33
**cTnI**	0.261	0.087	0.352*	0.016*

The present study did not find a significant association between the severity of respiratory and digestive signs and CRP, Hp and cTnI levels. However, patients with everted laryngeal saccules had more severe respiratory signs (*p* = 0.02) and higher levels of CRP (*p* = 0.044). The presence of tracheal hypoplasia was not associated with an increased severity of respiratory signs.

There was a significant correlation between the presence of digestive signs and cTnI (*p* = 0.016). When the severity digestive signs was studied in relation to cTnI levels, it was observed that animals with grade 2 of digestive signs had higher levels of cTnI than animals with grade 1 (*p* = 0.026). The possible relation of heart rate and arrhythmias with CRP, Hp and cTnI was statistically studied without obtaining any significance. In the same way, VHS values were not statistically associated with any biomarker (CRP, Hp and cTnI) or ECG values.

## Discussion

To the authors’ knowledge, this is the first prospective clinical study of dogs suffering from BAOS that has evaluated the levels of several biomarkers [acute phase proteins (CRP, Hp) and cTnI] and the relation of them with clinical signs and anatomical features.

The English bulldog has snoring and fragmented sleep [23] with mild hypoxemia and apneas only during rapid eye movement. English bulldog is the closest animal model for OSAS. Even if, UA obstructive disease in dogs is not equal to OSAS, important similarities exist. Some studies used brachycephalic dogs, as an animal model, to evaluate pharyngeal dilator muscular damages in UA obstructive disease, observing similar alterations to human patients with OSAS [23,24].

The correlation between human OSAS, inflammation and cardiovascular disease is well established [10,13,16]. The mechanism involved in the development of cardiovascular disease is attributed to increased sympathetic activity due to sleep deprivation and fragmentation, and repetitive hypoxemic events with significant arterial desaturation. These factors are believed to lead to an activation of pro-inflammatory cytokines. Even if OSAS and BAOS are different syndromes, this study tries to clear up a possible association of BAOS, inflammation and cardiovascular diseases.

The presence of digestive signs in dogs with BAOS has been described elsewhere [3,25-27]. The relationship between respiratory and digestive signs is due to the exaggerated repetitive variation of diaphragmatic pressure, present in patients with obstructive breathing, which causes gastroesophageal reflux and inflammation of the oropharyngeal areas that, in turn, worsen respiratory signs [3,27]. Poncet and others [3,27] found an association between the severity of respiratory and digestive signs in dogs with BAOS, which suggests that these signs are related. These authors also used endoscopy and histological studies to reveal the presence of lesions in the digestive tract of all dogs with BAOS. The majority of dogs with BAOS in the present study had digestive signs, but no association between the severity of digestive and respiratory signs was found. Even though the physiological relation of respiratory and digestive signs is clear, a correlation with severity of both signs is not always present. The results of the study indicate a correlation between the presence of digestive signs and high cTnI values, but authors did not find any plausible explanation for this association. Further studies should be performed in order to find the physiological reason of this correlation.

The most common anomalies present in dogs from the present study were stenotic nares and elongated soft palate, similar to findings described by previous studies [16,26,28]. However, the prevalence of everted laryngeal saccules in dogs of the present study was lower that previously reported [4,6,29]. Due to the prospective nature of this study, the presence of tracheal hypoplasia was evaluated in nearly all the cases included (96%) and was found in 14 dogs (29.1%). Tracheal hypoplasia is a concurrent finding in many patients with BAOS [1-3,5-7,25,30]. English bulldogs are more commonly affected by tracheal hypoplasia than other breeds, which could have biased our results [1,30,31]. But, according other studies and our results, in the absence of concurrent pulmonary disease, tracheal hypoplasia is usually not associated with clinical signs [31-33]. It may exacerbate the respiratory signs associated with BAOS because of increased resistance to airflow, but tracheal hypoplasia, according our results and other reports, is not associated with worse outcomes after surgical correction of BAOS [1,6,31,33,34].

The presence of nasopharyngeal turbinates can be an important anatomic component of BAOS [4], but in the present study it has not been evaluated in all dogs due to the multicentric nature of the study.

The innovative aspects of this work are the determination of different biomarkers in dogs with BAOS. The design of the study included a complete work-up to exclude other diseases that might affect the results of biomarker determination. In human medicine, high values of C-reactive protein (CRP) have been found in OSAS patients and have been associated with the severity of respiratory signs according to the apnea/hypopnea index [35]. The apnea/hypopnea index, obtained during polisomnography, is an important parameter for describing the severity of OSAS and describes the number of apneas or hypopneas per hour. Moreover levels of CRP, Hp and IL-6 in human patients with OSAS decreased after surgical or medical treatment [16,36]. A study performed with 17 brachycephalic dogs showed that some proinflammatory cytokines and nitric oxide are increased in brachycephalic dogs and also correlated with the severity of clinical signs [37]. Even though, in the present study few dogs had high values of CRP and Hp, and no correlation with the severity of respiratory signs was observed, with the exception of patients with everted laryngeal saccules. The presence of everted laryngeal saccules is considered a secondary change from airway obstruction, but can contribute to aggravate obstructive upper airway signs. A significant association has been found between the presence of everted laryngeal, severity of respiratory signs and CRP levels indicating that the presence of everted laryngeals can increase upper airway obstruction leading to severe respiratory signs and increase of inflammatory biomarkers. By contrast, the present results suggest that CRP and Hp determination, in general, do not offer valuable information in dogs with BAOS. Probably, in brachycephalic dogs, BAOS do not cause such an inflammatory status as OSAS in human patients. In human medicine, OSAS is diagnosed in adult patients and anatomical abnormalities are not the unique etiology of the syndrome. On the other hand, BAOS is diagnosed in young brachycephalic patients with upper airway abnormalities. Maybe those differences justify a less obvious systemic inflammatory response in dogs suffering from BAOS. Actually, a clinical grade system and upper airway examination can define BAOS in canine patients. Even though, an objective clinical test, such as apnea-hipopnea index, obtained during polisomnography and used to define human OSAS severity, could offer valuable information to better classify and understand BAOS. On the other hand, the respiratory functional assessment using barometric whole-body plethysmography in brachycephalic dogs could also be used to characterize the respiratory compromise in those dogs [5]. Due to the complexity of these tests, polisomnography and plethysmography were not available for our study.

Several authors suggested that cTnI determination in dogs is useful for identifying myocardial damage [38,39]. A large percentage of brachycephalic dogs (47.8%) had high levels of cTnI. It is interesting to note that all (100%) English bulldogs and 53.8% of French bulldog had high levels of cTnI. The hypothesis is that, as occurs in humans with OSAS, BAOS can induce surges in sympathetic activity, hypoxia and increased blood pressure that lead to myocardial damage.

All patients included in the study were submitted to a thorough physical examination, blood work, thoracic x-ray and an electrocardiogram, and no evidence of cardiac disease was found. However brachycephalic dogs may be predisposed to pulmonary hypertension, due to chronic hypoxia, and dogs with pulmonary hypertension can have increased concentrations of cTnI [40]. A limitation of the study due to its multicentric nature is that echocardiography was not performed in all dogs and therefore pulmonary hypertension and occult cardiac disease cannot be completely ruled out as a cause of increase cTnI in some dogs from this study. Even though, the evaluation of ECG (presence of arrhythmias and tachycardia) and vertebral heart score (VHS) was evaluated in relation to cTnI, CRP and Hp without obtaining any association. Moreover cTnI levels can vary with age and breed [38,41,42] and canine reference intervals must be established for different age groups and breeds. This information should be added to future studies. Although further studies must clarify this condition, the results obtained from this study suggest that some patients with BAOS had hidden but biochemically detectable myocardial injury. Even though, it must be considered that repeated measurements of those biomarkers levels in each individual dog could have increased the robustness of the results. On the other hand, the lack of a control group is an important limitation of the study since could have helped to interpret the results. But it must be taken to account, that is very difficult to find brachycephalic dogs with a normal breathing pattern because nearly all of them have some degree of upper airway obstruction.

## Conclusions

To conclude, this study suggests that BAOS does not induce an evident systemic inflammatory status according to values of CRP and Hp. On the other hand, these preliminary observations suggest that some dogs with BAOS may suffer some grade of myocardial damage that could be detected by cTnI levels. Further studies including more animals are required to define the predictive value of those biomarkers in dogs with BAOS. To the authors’ knowledge, this is the first description of CRP, Hp and cTnI levels in dogs with BAOS.

## Methods

This multicentre study is based on a prospective evaluation of brachycephalic dogs over a two-year period (January 2008–January 2010) in four veterinary hospitals (Hospital Clinic Veterinari, Barcelona; ARS Veterinaria, Barcelona; Centro Policlínico Veterinario El Raspeig, Alicante and Centre Hospitalier Vétérinaire Frégis, Arcueil). The study group consisted of brachycephalic dogs presented for the diagnosis of BAOS. In order to be included in the study dogs must be brachycephalic with clinical signs of upper airway obstruction, with a body condition from 4/9 to 7/9, without any previous treatment for BAOS and any present medication. On the other hand, dogs included in the study must not present any other illness apart from BAOS.

### Ethical authority

The procedures conducted for this study were absolutely designed to improve the welfare of dogs. Moreover, owners were exhaustively informed of the blood work and exploratory procedures applied on each animal, and signed an authorization file of acceptance. On the other hand, the study was evaluated and accepted by the Ethical Committee on Animal and Human Research (CEEAH) of the Universitat Autònoma de Barcelona (UAB).

### Clinical evaluation

Upon admission, a physical examination, thoracic radiography and an electrocardiogram were performed on all patients. Moreover cell blood count, serum biochemistry and serum levels of CRP, Hp and cTnI were evaluated. A complete clinical form was obtained from each patient, designed to homogenize the information between centers. The frequency of upper respiratory signs (stertors, snoring, inspiratory effort, exercise intolerance and syncope) and digestive signs (ptyalism, regurgitation and vomiting) were recorded. On the basis of the frequency of each respiratory and digestive sign, a global classification of four grades was obtained: absent (grade 0), mild (grade 1), moderate (grade 2) and severe (grade 3).

Diagnosis of all anatomical components of BAOS was made via oropharyngeal and laryngeal examination and thoracic radiographs in anesthetized dogs. Premedication included 0.05 mg/kg acepromacine and 0.01 mg/kg buprenorfine intramuscularly. Anaesthesia was induced with propofol. A soft palate that extended beyond its contact with the epiglottis, or that extended caudally to distal poles of the tonsils, was considered redundant. BAOS diagnosis was carried out on the basis of both upper airway respiratory signs and anatomic abnormalities, as has been described elsewhere [25].

Lateral thoracic radiography aimed at measuring the vertebral heart score and ratio of the thoracic inlet (DTI) to the tracheal diameter (DT) was performed anesthetized animals in order to detect tracheal hypoplasia, defined as DTI/DT ratio < 0.16 [22,34]. The findings of physical examinations, cardiac auscultation, electrocardiographic and radiographic abnormalities, and blood tests provided no evidence of any other diseases in patients included in the study.

### Biomarker analysis

Blood samples were collected in 5 mL serum blood tubes and centrifuged within 30 minutes after collection. The serum obtained was separated and stored at -80 °C for biomarker analysis. Sample storage varied from 1 to 2 months. Samples were sent using dry ice and were thawed only once, at the time of analysis.

CRP concentration was measured using a human immunoturbidimetric assay^a^ that showed a correlation of 0.98 with a specific canine ELISA assay^b^ which has been validated for use in dogs [43]. A pooled canine serum sample with high concentration of CRP measured by the specific canine ELISA assay was used as standard. Hp concentrations were measured by a commercially available colorimetric method^c^, previously validated in dogs [43]. CRP and Hp levels were measured in serum using an automated biochemistry analyzer^d^ and had intra-run and inter-run coefficients of variation < 10%. cTnI was determined by the DPC Immulite, a previously validated assay that gives satisfactory imprecision values [19]. CRP, Hp and cTnI levels were classified as normal or high according to our laboratory reference values (normal values: CRP < 20 mg/L; Hp < 3 g/L; cTnI < 0.05 ng/ml).

### Statistical analysis

The statistical analysis was performed using the statistical software SPSS statistics^e^. A value of *p*<0.05 was used to determine significance. Data were tested for normality by Shapiro-Wilk and Kolmogorov-Smirnov tests. Most data did not follow a normal distribution and non-parametric tests were employed. A Spearman Rho test (Table 2) was used to search for a correlation between the degree of clinical signs and all the studied parameters. Two variables relating to the severity of clinical signs were introduced into the database: grade of respiratory signs and grade of digestive signs. Grades 1, 2 and 3 for respiratory signs were considered for analysis. All grades were used for digestive signs. The Kruskall-Wallis test was employed to compare values for a single variable between three or more groups and differences between groups were compared with rank sum (Mann–Whitney U) tests.

## Endnotes

^a^CRP OSR 6147 Olympus Life and Material Science Europe GmbH, Lismeehan, O’Callaghan’s Mills, Co. Clare, Ireland

^b^Tridelta Phase range canine CRP kit, Tridelta Development Ltd, Brey, Ireland

^c^Tridelta Phase range haptoglobin kit, Tridelta Development Ltd.

^d^Olympus 2700 Automatic Chemistry Analyzer, Olympus Europe GmbH, Hamburg, Germany

^e^SPSS 17.0 version, Chicago, IL, USA

## Abbreviations

BAOS: Brachycephalic upper airway obstructive syndrome; CRP: C-reactive protein; CTnI: Cardiac troponin I; DTI/DT: Thoracic inlet to tracheal diameter; ECG: Electrocardiogram; Hp: Haptoglobin; OSAS: Obstructive sleep apnea syndrome; VHS: Vertebral heart score.

## Competing interests

The authors declare that they have no competing interests.

## Authors’ contribution

The experiments were conceived by MP, RC and JL. JP participated in the design of the study and performed the statistical analysis. MDT, CB, CP and JMC have made substantial contributions on the acquisition of data used in the manuscript and have participated on the interpretation of data. JJC have made all the biomarker analysis of the animals studied. All authors contributed to, and approved the final manuscript.
